# Supplementary material for: Quantum state tomography of molecules by ultrafast diffraction
Source: Nat Commun. 2021 Sep 14;12:5441. doi: 10.1038/s41467-021-25770-6 (PMC8440554; doi:10.1038/s41467-021-25770-6)
Supplement: Supplementary file 1 — Supplementary Information [file 41467_2021_25770_MOESM1_ESM.pdf]

Supplementary Information for
Quantum state tomography of molecules by ultrafast diffraction

Ming Zhang*,[1] Shuqiao Zhang*,[1,*] Yanwei Xiong,[2] Hankai Zhang,[1]
Anatoly A. Ischenko,[3] Oriol Vendrell,[4] Xiaolong Dong,[1] Xiangxu Mu,[1]
Martin Centurion,[2] Haitan Xu,[5,6,†] R. J. Dwayne Miller,[7,‡] and Zheng Li[1,§]

[1]*State Key Laboratory for Mesoscopic Physics and*
Collaborative Innovation Center of Quantum Matter,
School of Physics, Peking University, Beijing 10087, China

[2]*Department of Physics and Astronomy,*
University of Nebraska–Lincoln, Lincoln, NE, USA.

[3]*Lomonosov Institute of Fine Chemical Technologies,*
RTU-MIREA - Russian Technological University,
Vernadskii Avenue 86, 119571 Moscow, Russia

[4]*Physikalisch-Chemisches Institut, Universität Heidelberg,*
Im Neuenheimer Feld 229, D-69120 Heidelberg, Germany

[5]*Shenzhen Institute for Quantum Science and Engineering,*
Southern University of Science and Technology, Shenzhen 518055, China

[6]*School of Physical Sciences, University of Science and Technology of China, Hefei 230026, China*

[7]*Departments of Chemistry and Physics,*
University of Toronto, Toronto, Ontario M5S 3H6, Canada

[*] These authors contributed equally to this work.

[†] xuht@sustech.edu.cn

[‡] dmiller@lphys.chem.utoronto.ca

[§] zheng.li@pku.edu.cn

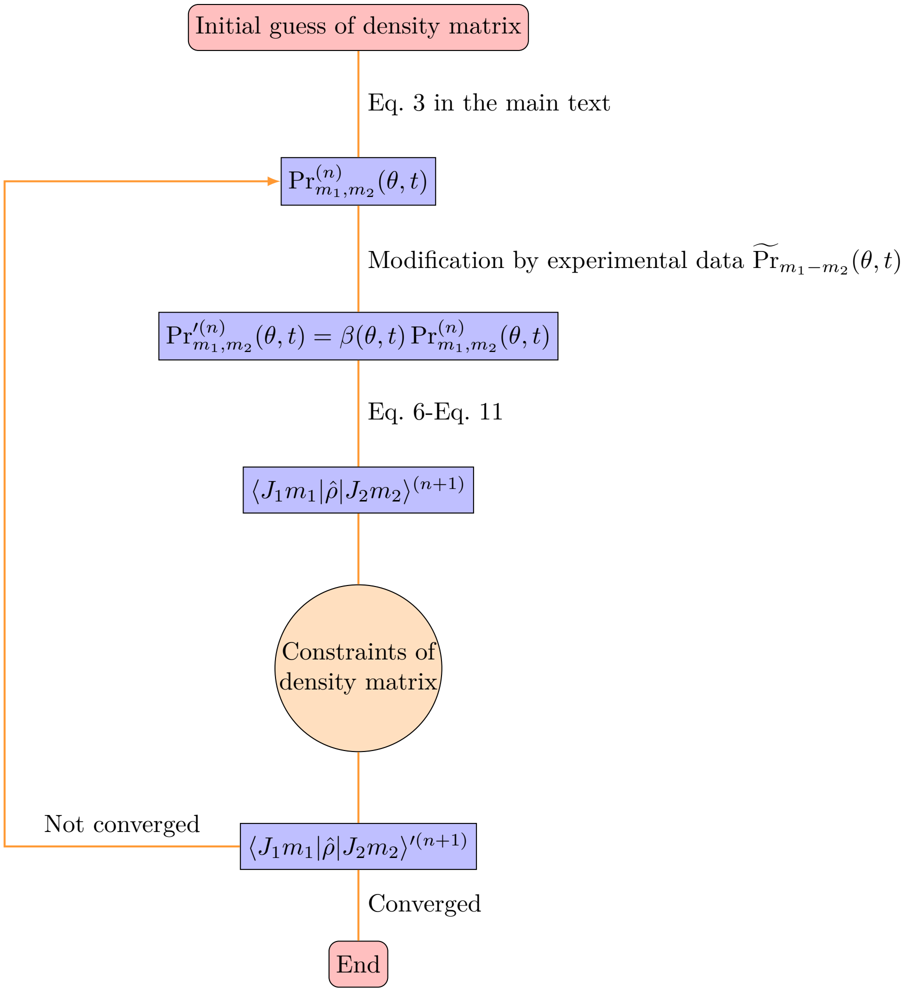

Supplementary Figure 1. Schematic flow chart for imposing constraints to the wavepacket probability distribution. The internal procedure for the ”constraints of density matrix” is separately elaborated in Supplementary Fig. 2. The superscript n represents n-th iteration.

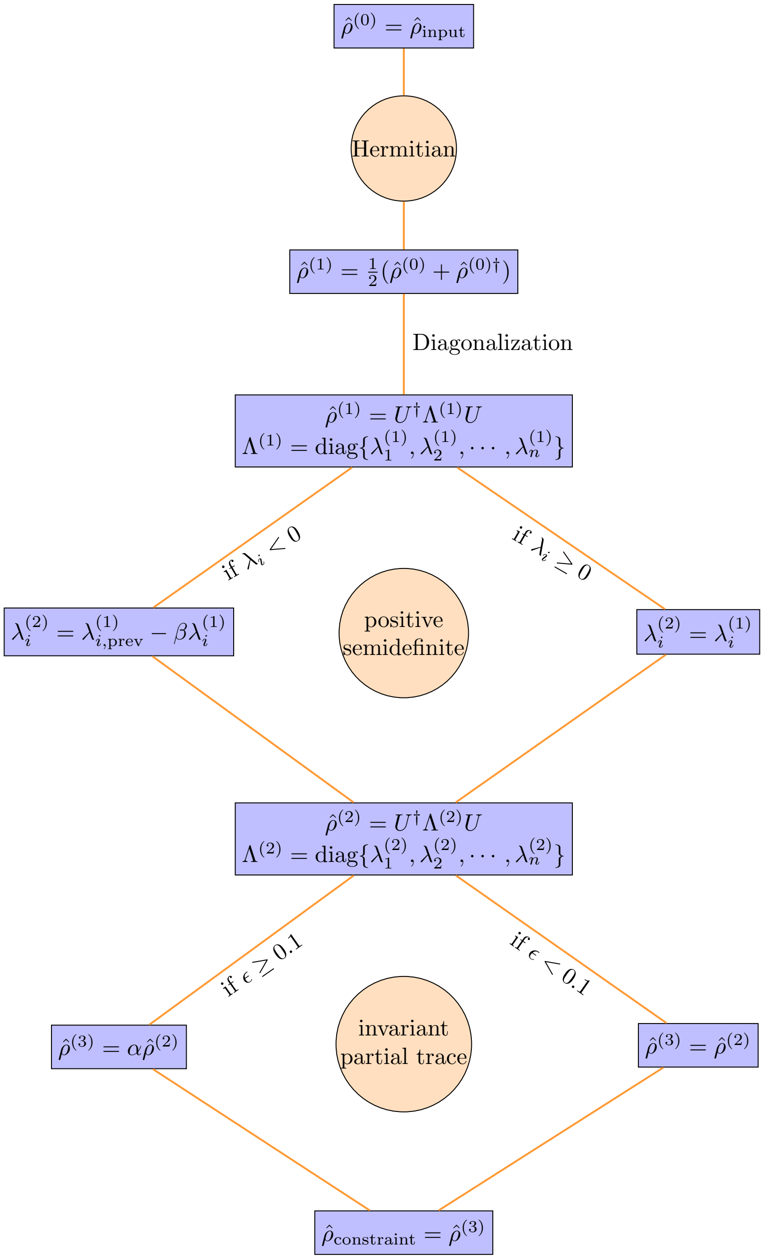

Supplementary Figure 2. Schematic flow chart for imposing constraints to the density matrix. Here $\epsilon = \left|\frac{\sum_{J_{\text{odd}}} - \sum_{J_{0\,\text{odd}}} \omega_{J_0}}{\sum_{J_{0\,\text{odd}}} \omega_{J_0}}\right|$. α is defined in Eq. 25. We use hybrid input-output (HIO) algorithm for the positivity constraint with $\beta = 0.9$ [1], where the subscript "prev" stands for the use of values in the previous iteration.

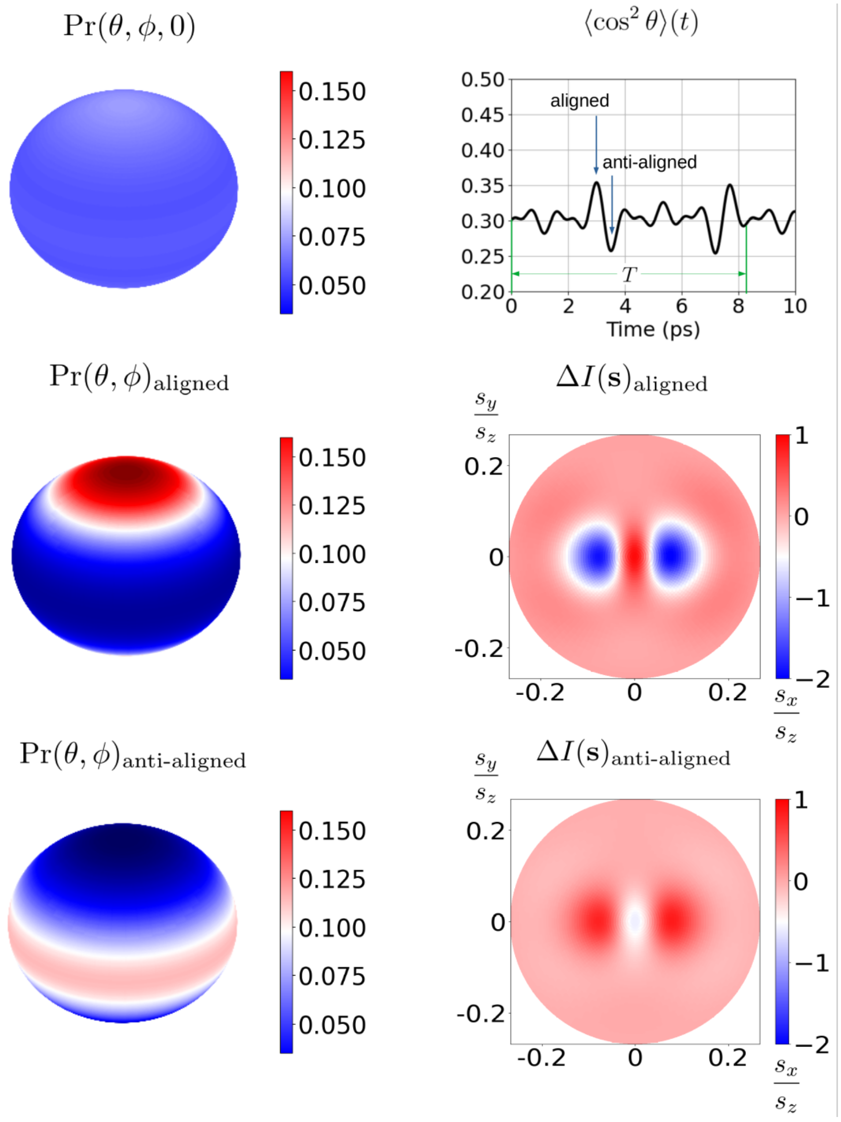

Supplementary Figure 3. Simulated probability distribution and diffraction pattern of rotational wavepacket. The first row shows the initial angular probability for N_2 molecules prepared at a rotational temperature of 30 K and the expectation values of $\cos^2\theta$ of the time evolving wavepacket for N_2 molecules after laser pulse [2]. The alignment laser pulse is linearly polarized with a Gaussian envelope of duration $\tau_L = 50$ fs and 10^{13} W/cm^2 peak intensity, and θ is the polar angle between the polarization and the molecular axes. The duration is much shorter than the characteristic rotational time $\tau_L \ll T$. The second and third rows show the angular probability distribution changes from aligned to anti-aligned, and the difference of their diffraction intensity with respect to $t = 0$. The X-ray photon energy is assumed to be 20 keV.

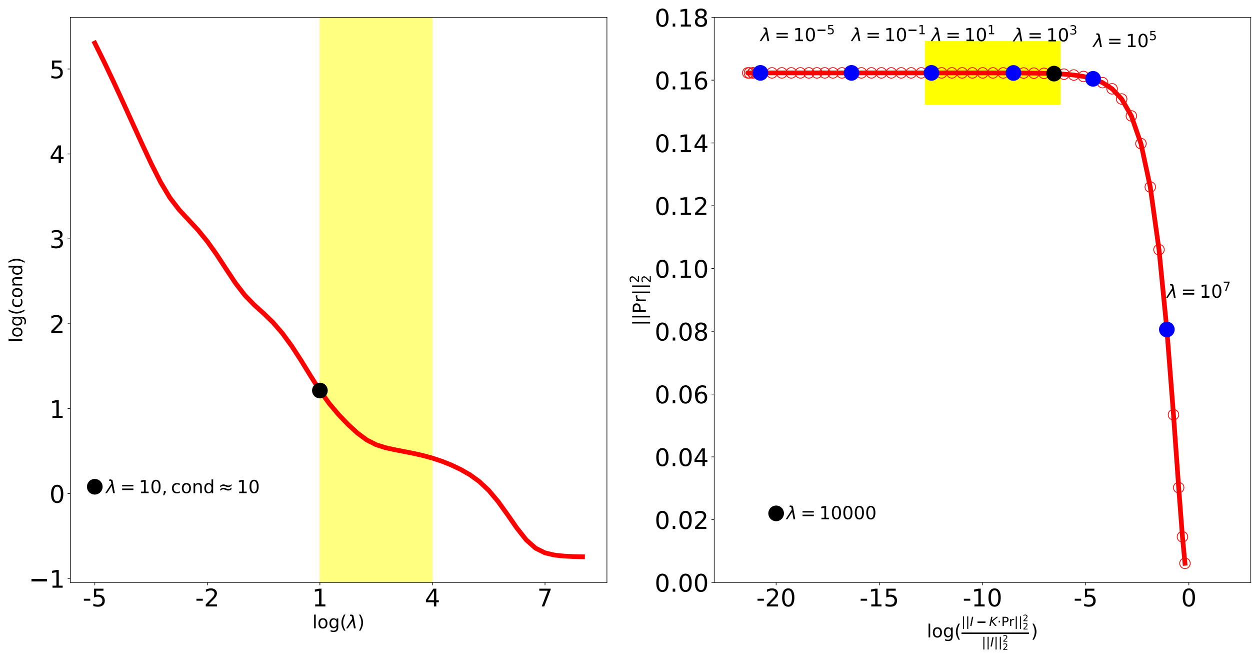

Supplementary Figure 4. Faithfulness of the probability distribution Pr obtained from integral equation with Tikhonov regularization. (Left) Logarithm of condition number versus logarithm of the regularization parameter λ. Larger λ makes the problem more insensitive to the measurement error ΔI. The approximate position of the black point marked on the sketch is (1,1) (we use an approximate position because every calculation that contains generation of the random numbers leads to slightly different curve). (Right) The values of $\|\mathrm{Pr}\|_2^2$ and the residual $\log(\frac{\|I-\mathbf{K}\cdot\mathrm{Pr}\|_2^2}{\|I\|_2^2})$ for λ ranging from 10^{-5} to 10^8. The Tikhonov regularization procedure minimizes $\|I-\mathbf{K}\mathrm{Pr}\|_2^2+\lambda\|\mathrm{Pr}\|_2^2$. The black point marked on the curve is the turning point corresponding to $\lambda \approx 10^4$. The yellow area starting from $\log\lambda = 1$ and ending at $\log\lambda = 4$ illustrates the admissible range of regularization parameter λ.

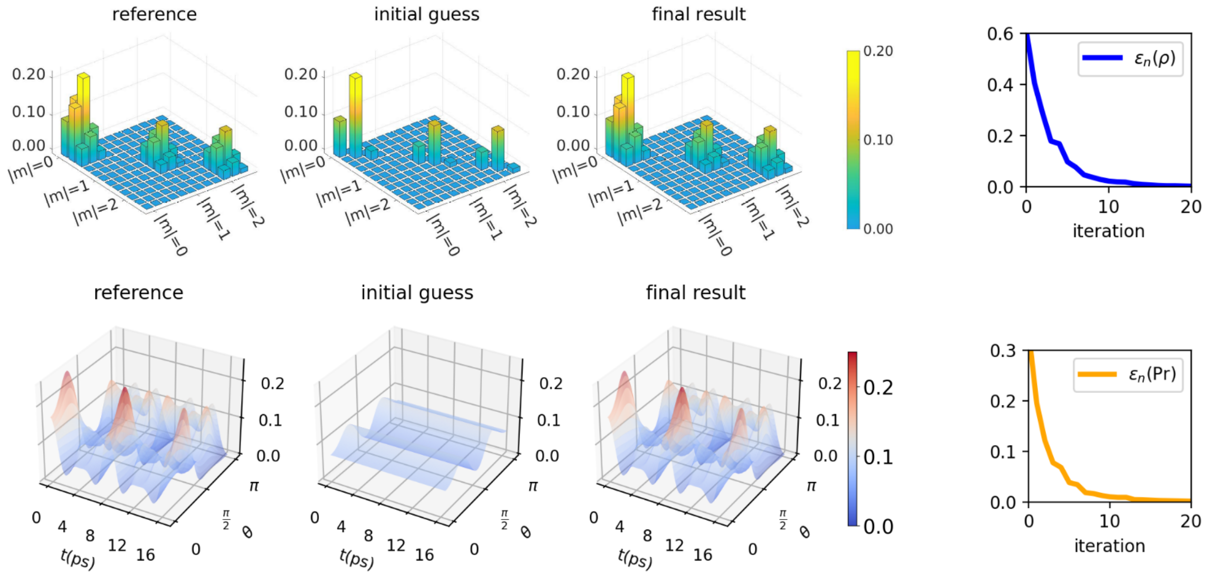

Supplementary Figure 5. Quantum tomography result of numerical trial with initial guess of correct diagonal elements of density matrix. The modulus of density matrix elements are shown in the upper panel, where $J = |m|, |m|+1, \cdots, J_{\max}$ within each m-block. The phases of all density matrix elements are zero at $t = 0$. The lower panel shows angular probability distribution, the recovered modulus and phases of density matrix elements faithfully reproduce the reference $\Pr(\theta, t)$, which is cylindrically symmetric in azimuthal direction of ϕ. Error functions of density matrix and probability distribution are shown in the rightmost column.

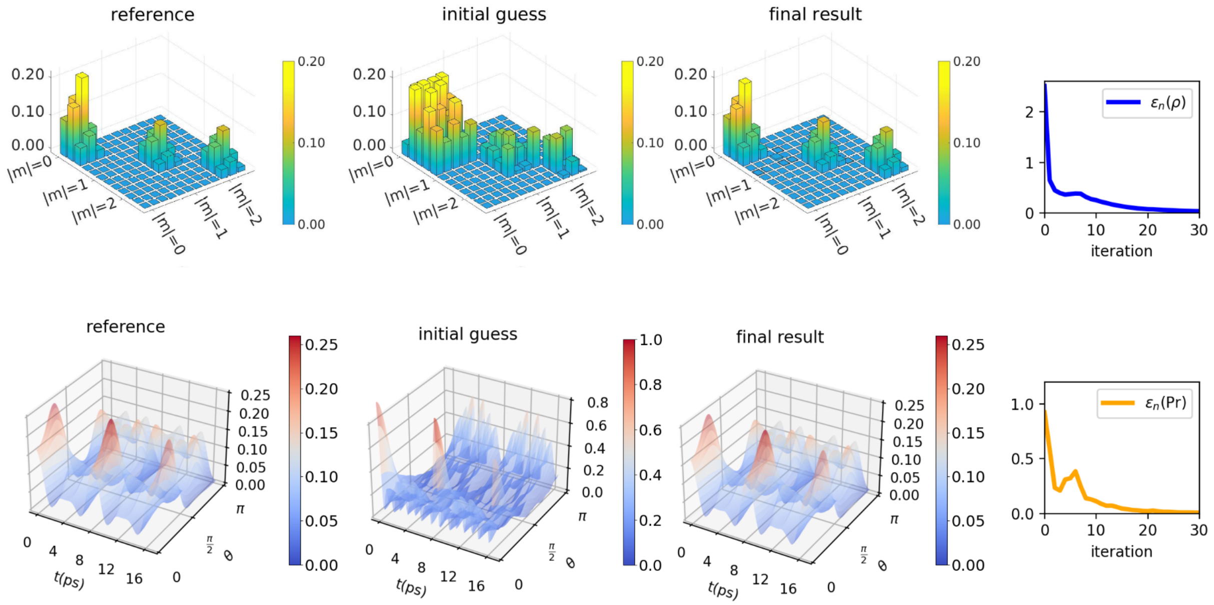

Supplementary Figure 6. Quantum tomography result of numerical trial with random initial guess of density matrix. Only the measured probability distribution and general properties of density matrix (being Hermitian, positive semidefinite and with unity trace) are imposed as constraints during the iteration algorithm. The density matrix to be recovered and its probability distribution are identical to that in Fig. 5. The modulus of density matrix elements are shown in the upper panel, where $J = |m|, |m|+1, \cdots, J_{\max}$ within each m-block. The phases of all density matrix elements are zero at $t = 0$. The lower panel shows angular probability distribution, the recovered modulus and phases of density matrix elements faithfully reproduce the reference $\mathrm{Pr}(\theta, t)$ (cylindrically symmetric in azimuthal direction of ϕ). Error functions of density matrix and probability distribution are shown in the rightmost column.

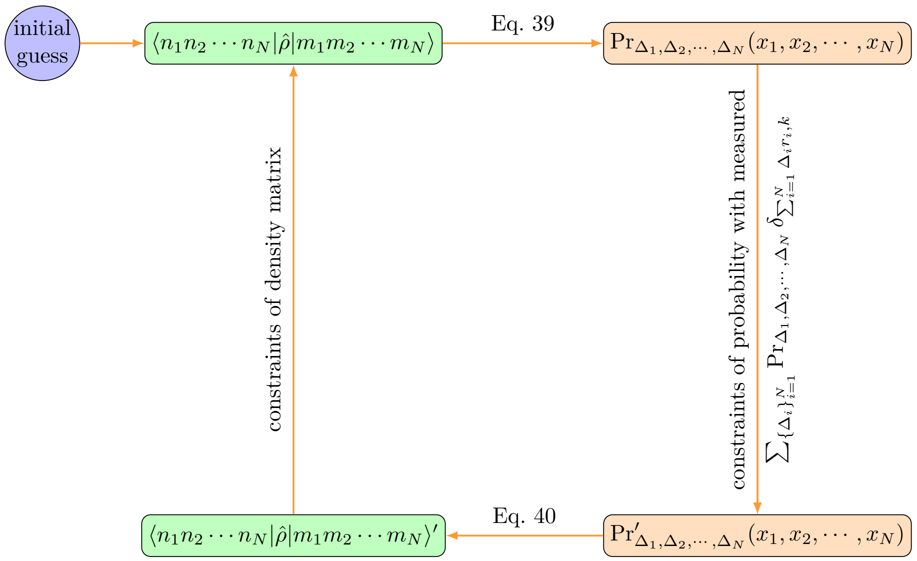

Supplementary Figure 7. Quantum tomography of vibrational state. The iterative transform is again between the spaces of density matrix and the blockwise probability distribution $\mathrm{Pr}_{\Delta_1,\Delta_2,\cdots,\Delta_N}(x_1, x_2, \cdots, x_N)$.

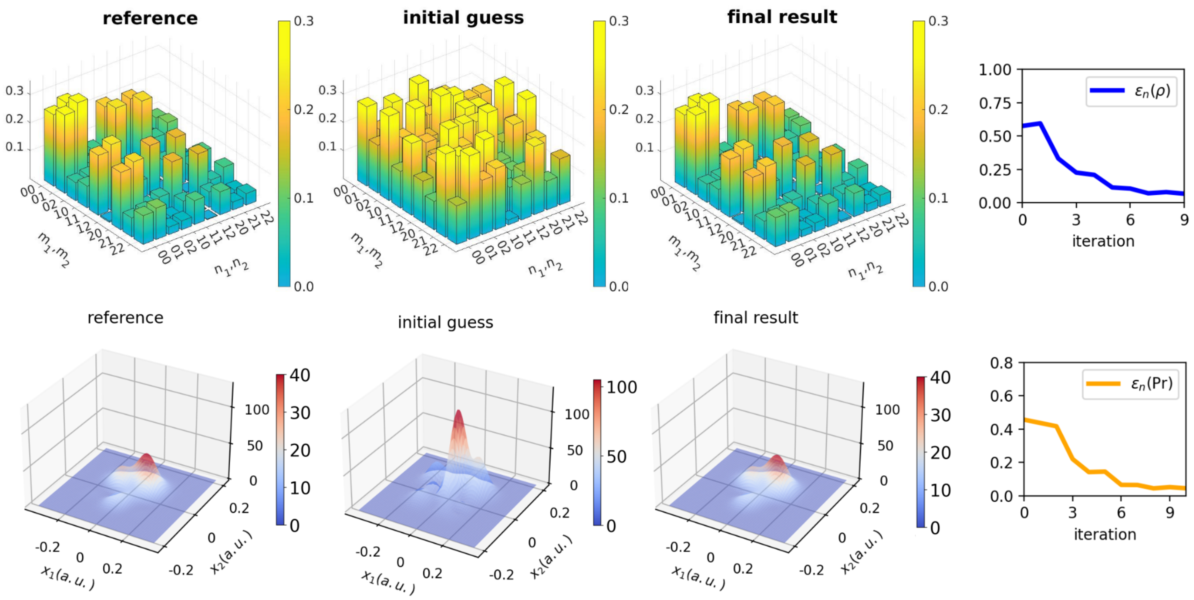

Supplementary Figure 8. Quantum tomography of two-dimensional vibrational wavepacket to the second order. The calculation is performed with reduced mass 12 amu, frequency $\omega_0 = 1209.8\,\mathrm{cm}^{-1}$ (0.15 eV) and frequency ratio of two vibrational modes $r_1/r_2 = 1/3$. The modulus of density matrix elements and probability distribution for a given time $t = 1.8$ fs are shown in the upper panel and lower panel, the recovered modulus and phases of density matrix elements faithfully reproduce the reference $\mathrm{Pr}(x_1, x_2, t)$. The algorithm converged for about 10 iterations as illustrated in the rightmost column, where $\epsilon_{10}(\hat{\rho}) = 4.1 \times 10^{-2}$ and $\epsilon_{10}(\mathrm{Pr}) = 3.1 \times 10^{-2}$.

SUPPLEMENTARY NOTE 1. EXPERIMENTAL DATA COLLECTION

The details of the keV UED setup and experimental conditions for nitrogen alignment experiment have been previously introduced in [3, 4]. We use a tilted infrared laser pulse to excite the rotational wave packet of the nitrogen molecule ensemble with a laser pulse duration of 60 fs, a spot size of 190 um (horizontal) × 260 um (vertical), and pulse energy of 1mJ. The tilted angle is about 60 degrees, which is designed to remove the group velocity mismatch due to the lower speed (0.526c, where c is the speed of light) of the electron pulse. The probe electron pulse is generated by shinning a 266 nm UV laser onto a copper cathode, which is accelerated by a 90 keV DC voltage and then compressed by a 3GHz RF electric field to minimize the temporal pulse duration on the sample. The electron beam is truncated using a platinum aperture with a diameter of 100 um to deliver a beam current of 8 pA, corresponding to 10,000 electrons per pulse. A de Laval nozzle with an inner diameter of 30 um is used to deliver the nitrogen molecules to the interaction as a supersonic molecular beam with a diameter of 200 um, and the nozzle backing pressure is 1200 mbar of nitrogen. The instrument response time was determined to be 240 fs by fitting the experimental anisotropy to its corresponding simulation. The timing jitter was 50 fs rms over several hours[4]. The electron diffraction patterns are recorded by an electron-multiplying charge-coupled device (EMCCD) camera, and the time delay between the pump and probe is controlled by an optical stage. Here the step of time delay is 100 fs.

SUPPLEMENTARY NOTE 2. DIFFRACTION PATTERN TREATMENT

The details of how to retrieve the angular distribution from the measured diffraction patterns have been explained in [4]. Briefly, the diffraction difference pattern for each image is calculated with $\Delta I(\mathbf{s},t) = I(\mathbf{s},t) - I(\mathbf{s},t < -1\text{ps})$ to remove the background of atomic scattering, and then are averaged over the four quadrants using its symmetry. The simulated random molecular scattering with a rescaling factor of 0.35, which is obtained by fitting the experimental anisotropy evolution and its corresponding simulation, is added to $\Delta I(\mathbf{s},t)$ to recover molecular diffraction intensity $I(\mathbf{s},t)$. The modified pair distribution function (MPDF) [4] is calculated by applying the inverse Fourier transform of $I(\mathbf{s},t)$, followed by an Abel inversion, giving the information of angular distribution $\Pr(\theta,\phi,t)$.

The angular distribution retrieved from experimental data covers the initial alignment through

the revivals up to about 7 ps, which is deconvolved using the algorithm in [5–7]. The instrumental point spread function (PSF) is assumed to be a one-dimensional Gaussian function with a full width at half maximum of 0.28 ps, corresponding to the general temporal resolution of the setup, for the deconvolution, which eliminates the blurring due to the limit temporal resolution of the setup. The general temporal resolution 0.28 ps is obtained by comparing the deconvoluted angular distribution evolution with the simulation and minimizing their difference. The temporal evolution of $\Pr(\theta, \phi, t)$ is extended to obtain the data up to 11ps by a reflection of the angular distribution evolution from 6.1ps to 1.2 ps to approximate the data from 6.1 ps to 11 ps according to the approximate symmetry based on the simulation.

SUPPLEMENTARY NOTE 3. QUANTUM TOMOGRAPHY FOR STATES IN m-BLOCK WITH FIXED PROJECTION QUANTUM NUMBERS

We extend the treatment in Ref. [8] to show that the density matrix element $\langle J_1 m_1|\hat{\rho}|J_2 m_2\rangle$ in the (m_1, m_2)-block subspace can be solved analytically, once the blockwise probability density $\Pr_{m_1,m_2}(\theta, t)$ of given projection quantum numbers m_1, m_2 is determined. We expand the blockwise probability density with eigenbasis,

$$\Pr_{m_1,m_2}(\theta, t) = \sum_{J_1=|m_1|}^{\infty} \sum_{J_2=|m_2|}^{\infty} \langle J_1 m_1|\hat{\rho}|J_2 m_2\rangle \widetilde{P}_{J_1}^{m_1}(\cos\theta) \widetilde{P}_{J_2}^{m_2}(\cos\theta) e^{-i\Delta\omega t}\,, \tag{1}$$

where the energy level difference is

$$\Delta\omega = \omega_{J_1} - \omega_{J_2} = \frac{\Delta J(J+1)}{2\mathcal{I}}\,,$$

$\Delta J = J_1 - J_2$, $J = J_1 + J_2$ and $\mathcal{I}$ is the moment of inertia of the rotating molecule. For the sake of convenience, we define normalized associated Legendre polynomials

$$\widetilde{P}_J^m(\cos\theta) = (-1)^m \sqrt{\frac{(2J+1)(J-m)!}{2(J+m)!}} P_J^m(\cos\theta)\,, \tag{2}$$

with orthonormal relations

$$\int_0^\pi \sin\theta d\theta \widetilde{P}_{J_1}^m(\cos\theta) \widetilde{P}_{J_2}^m(\cos\theta) = \delta_{J_1,J_2}\,. \tag{3}$$

We use the orthogonal relations of Legendre polynomials and exponential functions in the integral transformation [8]. Firstly, consider the motion along rotational polar coordinate θ. The product

of two associated Legendre polynomials occur in Eq. 1 can be expanded by single associated Legendre polynomials

$$\widetilde{P}_{J_1}^{m_1}(\cos\theta)\widetilde{P}_{J_2}^{m_2}(\cos\theta) = \sum_{L=|J_1-J_2|}^{J_1+J_2} C_{J_1m_1J_2m_2}^{L,m_1+m_2}\widetilde{P}_{L}^{m_1+m_2}(\cos\theta)\,, \tag{4}$$

$$C_{J_1m_1J_2m_2}^{L,m_1+m_2} = \sqrt{\frac{(2J_1+1)(2J_2+1)}{4\pi(2L+1)}}\langle J_1m_1J_2m_2|L(m_1+m_2)\rangle\langle J_10J_20|L0\rangle\,. \tag{5}$$

Thus, integrate over θ,

$$\begin{aligned} I_{m_1m_2}(\alpha,t) &= \int_0^\pi \sin\theta d\theta \widetilde{P}_{\alpha}^{m_1+m_2}(\cos\theta)\mathrm{Pr}_{m_1,m_2}(\theta,t) \\ &= \sum_{J_1=|m_1|}^{\infty}\sum_{J_2=|m_2|}^{\infty}\sum_{L=|\Delta J|}^{J} C_{J_1m_1J_2m_2}^{L,m_1+m_2}\langle J_1m_1|\hat{\rho}|J_2m_2\rangle e^{-i\Delta\omega t} \\ &\quad\times\int_0^\pi \sin\theta d\theta\widetilde{P}_{\alpha}^{m_1+m_2}(\cos\theta)\widetilde{P}_{L}^{m_1+m_2}(\cos\theta) \\ &= \sum_{J_1=|m_1|}^{\infty}\sum_{J_2=|m_2|}^{\infty} C_{J_1m_1J_2m_2}^{\alpha,m_1+m_2}\langle J_1m_1|\hat{\rho}|J_2m_2\rangle e^{-i\Delta\omega t}\,. \end{aligned} \tag{6}$$

Let $T = 2\pi\mathcal{I}$, which is the rotational revival period [9], and integrate over t,

$$\begin{aligned} I_{m_1m_2}(\alpha,\beta) &= \frac{1}{2T}\int_0^{2T} I_{m_1m_2}(\alpha,t)e^{i\beta(\alpha+1)t/2\mathcal{I}}dt \\ &= \sum_{J_1=|m_1|}^{\infty}\sum_{J_2=|m_2|}^{\infty} C_{J_1m_1J_2m_2}^{\alpha,m_1+m_2}\langle J_1m_1|\hat{\rho}|J_2m_2\rangle\delta_{\beta(\alpha+1)-\Delta J(J+1)}\,. \end{aligned} \tag{7}$$

where the range of integration $2T$ is the minimal period of $e^{i[\beta(\alpha+1)/2\mathcal{I}-\Delta\omega]t}$ because

$$\left[\beta(\alpha+1)/2\mathcal{I}-\Delta\omega\right]2T = 2\pi\left[\beta(\alpha+1)-\Delta J(J+1)\right]\,, \tag{8}$$

which is the integral multiple of 2π. The range of α and β is set to be $|\Delta J| \leq |\beta| \leq \alpha \leq J$, where β and ΔJ are of the same sign. If $\beta(\alpha+1)$ has unique integer factorization, the only term remaining in the sum satisfying

$$\beta(\alpha+1) = \Delta J(J+1) \tag{9}$$

is $\beta = \Delta J$ and $\alpha = J$. The corresponding density matrix element can be derived as

$$\langle\frac{\alpha+\beta}{2}m_1|\hat{\rho}|\frac{\alpha-\beta}{2}m_2\rangle = \frac{I_{m_1m_2}(\alpha,\beta)}{C_{\frac{\alpha+\beta}{2}m_1\frac{\alpha-\beta}{2}m_2}^{\alpha,m_1+m_2}}\,. \tag{10}$$

If the factorization of $\beta(\alpha+1)$ is not unique, we calculate all integrations $I_{m_1m_2}(\alpha', \beta')$ where $\beta(\alpha+1) = \beta'(\alpha'+1)$. For example, when $\beta = 0$,

$$I_{m_1m_2}(\alpha, 0) = \sum_{J=\max\{|m_1|,|m_2|\}}^{\infty} C^{\alpha, m_1+m_2}_{Jm_1Jm_2} \langle Jm_1|\hat{\rho}|Jm_2\rangle \tag{11}$$

all of the $\Delta J = 0$ terms remain. When changing the value of α, all these $I_{m_1m_2}$ and corresponding density matrix elements constitute a set of linear algebraic equations (where $\alpha = 2J$ can only be even numbers),

$$\begin{pmatrix} I_{m_1m_2}(\alpha,0) \\ I_{m_1m_2}(\alpha+2,0) \\ I_{m_1m_2}(\alpha+4,0) \\ \vdots \end{pmatrix} = \begin{pmatrix} C^{\alpha,m_1+m_2}_{\frac{\alpha}{2}m_1\frac{\alpha}{2}m_2} & C^{\alpha,m_1+m_2}_{\frac{\alpha}{2}+1,m_1,\frac{\alpha}{2}+1,m_2} & C^{\alpha,m_1+m_2}_{\frac{\alpha}{2}+2,m_1,\frac{\alpha}{2}+2,m_2} & \cdots \\ 0 & C^{\alpha+2,m_1+m_2}_{\frac{\alpha}{2}+1,m_1,\frac{\alpha}{2}+1,m_2} & C^{\alpha+2,m_1+m_2}_{\frac{\alpha}{2}+2,m_1,\frac{\alpha}{2}+2,m_2} & \cdots \\ 0 & 0 & C^{\alpha+4,m_1+m_2}_{\frac{\alpha}{2}+2,m_1,\frac{\alpha}{2}+2,m_2} & \cdots \\ \vdots & \vdots & \vdots & \cdots \end{pmatrix} \times \begin{pmatrix} \langle\frac{\alpha}{2}m_1|\hat{\rho}|\frac{\alpha}{2}m_2\rangle \\ \langle\frac{\alpha}{2}+1,m_1|\hat{\rho}|\frac{\alpha}{2}+1,m_2\rangle \\ \langle\frac{\alpha}{2}+2,m_1|\hat{\rho}|\frac{\alpha}{2}+2,m_2\rangle \\ \vdots \end{pmatrix}, \tag{12}$$

which has unique solution because all diagonal terms of the upper triangular matrix are nonzero.

SUPPLEMENTARY NOTE 4. LASER ALIGNMENT OF ROTATING MOLECULE

The effective Hamiltonian of rotating molecule-laser interaction is [2]

$$\begin{aligned} \hat{H}_{\text{eff}} &= \hat{H}_0 + \hat{H}_{\text{int}} \\ \hat{H}_0 &= B\mathbf{J}^2 \\ \hat{H}_{\text{int}} &= -\frac{1}{2}\epsilon^2(t)[(\alpha_\parallel - \alpha_\perp)\cos^2\theta + \alpha_\perp], \end{aligned} \tag{13}$$

where $\mathbf{J}$ is the rotational angular momentum, $\epsilon(t)$ is the electric field of the laser pulse, $B = 1/2\mathcal{I}$ is the rotational constant, $\alpha_\parallel$ and $\alpha_\perp$ are the components of the static polarizability, parallel and perpendicular to the molecular axes. The molecule is assumed to be in the vibrational and electronic ground state. An initial rotational eigenstate $|J_0M_0\rangle$ evolves to a pendular state [2]

$$|J_0m_0\rangle \to |\psi(t)\rangle^{(J_0m_0)} = \sum_J d_J^{(J_0m_0)}|Jm_0\rangle e^{-iE_Jt/\hbar}, \tag{14}$$

where J and J_0 are of the same parity. The coupling coefficients $d_J^{J_0m_0}$ is induced by laser field, satisfying selection rules $\Delta m = 0$ and $\Delta J = 0, \pm 2$. $d_J^{J_0m_0}$ is invariant after the laser pulse, and the evolution of rotational angular distribution originates from interference of each dynamical phase. The coherence of the created quantum state can be maintained for several revival periods, and the alignment is reconstructed at predetermined times and survives for a perfectly controllable period [2], the sufficiently long coherence time makes the time evolution measurement of quantum state tomography feasible.

The initial system in thermal equilibrium can be characterized by the following density operator

$$\hat{\rho}_{\text{ini}} = \sum_{J_0m_0} \omega_{J_0} |J_0m_0\rangle\langle J_0m_0| \,, \tag{15}$$

where ω_{J_0} is the Boltzmann statistical factor determined by the rotational temperature. The density operator of the laser-aligned system is

$$\begin{aligned}\hat{\rho}(t) &= \sum_{J_0m_0} \omega_{J_0} |\psi(t)^{(J_0m_0)}\rangle\langle\psi(t)^{(J_0m_0)}| \\ &= \sum_{m_0}\left[\sum_{J_0}\omega_{J_0}\left(\sum_{J_1} d_{J_1}^{(J_0m_0)}|J_1m_0\rangle\right)\left(\sum_{J_2} d_{J_2}^{*(J_0m_0)}\langle J_2m_0|\right)\right] e^{-i(E_{J_1}-E_{J_2})t/\hbar} \\ &= \sum_{J_1J_2m}\left(\sum_{J_0}\omega_{J_0} d_{J_1}^{(J_0m)} d_{J_2}^{*(J_0m)}\right) e^{-i(E_{J_1}-E_{J_2})t/\hbar}|J_1m\rangle\langle J_2m| \,.\end{aligned} \tag{16}$$

And its density matrix elements are

$$\langle J_1m_1|\hat{\rho}(t)|J_2m_2\rangle = \delta_{m_1m_2}\left(\sum_{J_0}\omega_{J_0} d_{J_1}^{(J_0m_1)} d_{J_2}^{*(J_0m_2)}\right) e^{-i(E_{J_1}-E_{J_2})t/\hbar} \,. \tag{17}$$

So the partial trace of m subspace with odd (or even) J is invariant in the dynamics of laser alignment, since it is a general property of laser-molecule interaction,

$$\sum_{J_{\text{odd}}}\langle Jm|\hat{\rho}|Jm\rangle = \sum_{J_{\text{odd}}}\sum_{J_{0\,\text{odd}}}\omega_{J_0}|d_J^{(J_0m)}(t)|^2 = \sum_{J_{0\,\text{odd}}}\omega_{J_0} \,, \tag{18}$$

where we used the normalization property of coefficients $d_J^{J_0M}(t)$ in Eq. 14.

Notice that density matrix of opposite magnetic quantum number m and $-m$ is symmetric for $\hat{\rho}_{\text{ini}}$, which also remains symmetric for transition matrix element induced by laser interaction $\hat{H}_{\text{eff}}(t)$. From Eq. 13, taking into account selection rule $\Delta M = 0$,

$$\begin{aligned}&\langle J_1m|\hat{H}_{\text{eff}}(t)|J_2m\rangle = \langle J_1,-m|\hat{H}_{\text{eff}}(t)|J_2,-m\rangle \\ &= \delta_{J_1,J_2}\left[BJ_1(J_1+1) - \frac{1}{2}\epsilon^2(t)\alpha_\perp\right] - \frac{1}{2}\epsilon^2(t)(\alpha_\parallel - \alpha_\perp)\langle J_1m|\cos^2\theta|J_2m\rangle \,,\end{aligned} \tag{19}$$

where $\langle J_1 m|\cos^2\theta|J_2 m\rangle = \langle J_1,-m|\cos^2\theta|J_2,-m\rangle$ according to the properties of Clebesh-Gordan coefficients. The coefficients of pendular state $d_J^{(J_0 m_0)}$, which are totally determined by initial condition $\hat{\rho}_{\rm ini}$ and the Schrödinger equation,

$$i\dot{d}_J^{(J_0 m)} = \sum_{J'} \langle Jm|\hat{H}_{\rm eff}(t)|J'm\rangle \,, \tag{20}$$

are also symmetric $d_J^{(J_0 m)} = d_J^{(J_0,-m)}$. So are the density matrix elements

$$\langle J_1 m_1|\hat{\rho}|J_2 m_2\rangle = \sum_{J_0} \omega_{J_0} d_{J_1}^{(J_0,m_1)} d_{J_2}^{*(J_0,m_2)} = \langle J_1,-m_1|\hat{\rho}|J_2,-m_2\rangle \,. \tag{21}$$

SUPPLEMENTARY NOTE 5. THE ALGORITHM FOR IMPOSING CONSTRAINTS OF ITERATIVE QUANTUM TOMOGRAPHY

In this section we show the detailed procedure for making an arbitrary density matrix and probability distribution to satisfy the physical constraints given in the main text. Most physical constraints are given in the summation form. For example, from Eq. 18,

$$\sum_{J_{\rm odd}} \langle Jm|\hat{\rho}|Jm\rangle = \sum_{J_{0\,\rm odd}} \omega_{J_0} \,. \tag{22}$$

From the measured probability distribution

$$\begin{aligned}
&\widetilde{\Pr}_{m_1-m_2}(\theta,t) = \int_0^{2\pi} d\phi \Pr(\theta,\phi,t) e^{-i(m_1-m_2)\phi} \qquad (23)\\
&= \frac{1}{2\pi} \sum_{J_1 m_1' J_2 m_2'} \langle J_1 m_1'|\hat{\rho}|J_2 m_2'\rangle \widetilde{P}_{J_1}^{m_1}(\cos\theta) \widetilde{P}_{J_2}^{m_2}(\cos\theta) e^{-i\Delta\omega t} \int_0^{2\pi} d\phi e^{im_1'\phi} e^{-im_2'\phi} e^{-i(m_1-m_2)\phi}\\
&= \sum_{m_1' m_2'} \delta_{m_1'-m_2',m_1-m_2} \sum_{J_1 J_2} \langle J_1 m_1'|\hat{\rho}|J_2 m_2'\rangle \widetilde{P}_{J_1}^{m_1}(\cos\theta) \widetilde{P}_{J_2}^{m_2}(\cos\theta) e^{-i\Delta\omega t} \,,
\end{aligned}$$

and the constraint can be expressed as

$$\sum_{m_1'-m_2'=m_1-m_2} \Pr_{m_1',m_2'}(\theta,t) = \widetilde{\Pr}_{m_1-m_2}(\theta,t) \,. \tag{24}$$

They can be sataisfied by scaling with a common factor

$$\langle Jm|\hat{\rho}|Jm\rangle \to \alpha \langle Jm|\hat{\rho}|Jm\rangle \,, \quad \alpha = \frac{\sum_{J_{0\,\rm odd}} \omega_{J_0}}{\sum_{J_{\rm odd}} \langle Jm|\hat{\rho}|Jm\rangle} \,. \tag{25}$$

$$\Pr_{m_1,m_2}(\theta,t) \to \beta(\theta,t) \Pr_{m_1,m_2}(\theta,t) \,, \quad \beta = \frac{\widetilde{\Pr}_{m_1-m_2}(\theta,t)}{\sum_{m_1'-m_2'=m_1-m_2} \Pr_{m_1',m_2'}(\theta,t)} \,. \tag{26}$$

The constraints in probability space is given by Eq. 26, and illustrated with flow chart in Fig. 1. Further constraints in density matrix space include being Hermitian, positive semidefinite and having invariant partial traces (the procedure is presented with the flow chart in Fig. 2).

As a general rule to guarantee the completeness of constraint conditions, we can firstly analyse the physical system and find out the possible states, which could give same probability distribution for all time and are indistinguishable without further constraint, and construct the set of physical conditions that can distinguish the states from each other, e.g. selection rules, symmetry. The obtained physical conditions can be then used as constraints in the iterative QT procedure. In this manner, the completeness of the constraint conditions and the faithfulness of the converged density matrix solution can be achieved, i.e. the converged solution of the inversion problem is the true density matrix of the physical system.

SUPPLEMENTARY NOTE 6. BENCHMARKING ITERATIVE QUANTUM TOMOGRAPHY WITH SIMULATED ULTRAFAST DIFFRACTION OF N_2 ROTATIONAL WAVEPACKET

We use the new QT method to extract rotational density matrix from simulated ultrafast diffraction dataset of impulsively aligned nitrogen molecule, prepared at rotational temperature of 30 K. As shown in Fig. 3, from a simulated dataset consisting of a series of time-ordered snapshots of diffraction patterns [10]

$$I(\mathbf{s},t) = \int_0^{2\pi} d\phi \int_0^{\pi} \sin\theta d\theta \mathrm{Pr}(\theta,\phi,t)|f(\mathbf{s},\theta,\phi)|^2 , \quad (27)$$

the time-dependent molecular probability distribution $\mathrm{Pr}(\theta,\phi,t)$ can be obtained by solving the Fredholm integral equation of the first kind using Tikhonov regularization procedure [11]. We assume $\tau = -\cos\theta$ and replace the integral by Riemann summation,

$$I(\Theta_k,\Phi_l) = \sum_{i=1}^{a} \Delta\phi \sum_{j=1}^{b} \Delta\tau |f(\phi_i,\theta(\tau_j),\Theta_k,\Phi_l)|^2 \mathrm{Pr}(\phi_i,\theta(\tau_j)) , \quad (28)$$

at each instant, where $\Delta\phi = \frac{2\pi}{a}$, $\Delta\tau = \frac{2}{b}$, i is ranging from 1 to a, j is ranging from 1 to b, k is ranging from 1 to c, and l is ranging from 1 to d. ϕ and θ are the azimuthal and levitation angles of the linear molecular rotor, Θ and Φ are the scattering angle of the X-ray photon in the lab system (as is shown in Fig. 1 in the main text). We can write the total diffraction intensity in the matrix

form $I = \mathbf{K}\mathrm{Pr}$, where

$$I = \begin{pmatrix} I(\Theta_1, \Phi_1) \\ \vdots \\ I(\Theta_1, \Phi_d) \\ I(\Theta_2, \Phi_1) \\ \vdots \\ I(\Theta_c, \Phi_d) \end{pmatrix},$$

$$\mathbf{K} = \begin{pmatrix} |f(\phi_1, \theta_1, \Theta_1, \Phi_1)|^2 \Delta\phi\Delta\tau & \cdots & |f(\phi_a, \theta_b, \Theta_1, \Phi_1)|^2 \Delta\phi\Delta\tau \\ \vdots & \ddots & \vdots \\ |f(\phi_1, \theta_1, \Theta_c, \Phi_d)|^2 \Delta\phi\Delta\tau & \cdots & |f(\phi_a, \theta_b, \Theta_c, \Phi_d)|^2 \Delta\phi\Delta\tau \end{pmatrix},$$

$$\mathrm{Pr} = \begin{pmatrix} \mathrm{Pr}(\phi_1, \theta_1) \\ \vdots \\ \mathrm{Pr}(\phi_1, \theta_b) \\ \mathrm{Pr}(\phi_2, \theta_1) \\ \vdots \\ \mathrm{Pr}(\phi_a, \theta_b) \end{pmatrix}. \tag{29}$$

To avoid singular matrix inversion, we use Tikhonov regularization to get the rotational probability distribution,

$$\mathrm{Pr} = (\mathbf{K}^T\mathbf{K} + \lambda\mathbf{E})^{-1}\mathbf{K}^T I\,, \tag{30}$$

where $\mathbf{E}$ is identity matrix of size $(c \times d)$ and $\mathbf{K}^T$ is the transpose of matrix $\mathbf{K}$.

The Tikhonov regularization performs excellently in dealing with experimental data with measurement errors and preventing overfitting, and can faithfully recover the probability density distribution. To validate the faithfulness of the obtained probability distribution $\mathrm{Pr}(\theta, \phi)$, we define the condition number

$$\mathrm{cond} = \frac{\|\Delta\mathrm{Pr}\|_2/\|\mathrm{Pr}\|_2}{\|\Delta I\|_2/\|I\|_2}\,, \tag{31}$$

where $\|A\|_2 = \sqrt{\sum_i A_i^2}$ is the $\mathcal{L}^2$ Euclid norm. The condition number characterizes the degree of variation of the solution $\mathrm{Pr}(\theta, \phi)$ with respect to the input data of measured diffraction intensity $I(\mathbf{s})$, its value provides a measure for the sensitivity of the solution with respect to the measurement error and choice of regularization parameters. From Fig. 4, we can estimate that $\lambda \geq 10$ is required to ensure $\mathrm{cond} \leq 10$, and subsequently to ensure the reliability of the solution.

Quantum tomography of the rotational wavepacket gives the result shown in Fig. 3 in the main text. After 50 iterations, both density matrix and probability distribution are precisely recovered. The error of density matrix is $\epsilon_{50}(\hat{\rho}) = 2.9 \times 10^{-2}$ and error of probability achieves $\epsilon_{50}(\mathrm{Pr}) = 3.8 \times 10^{-5}$.

SUPPLEMENTARY NOTE 7. NUMERICAL TRIAL WITH RANDOMLY CHOSEN DENSITY MATRIX AND INITIAL GUESS

We have verified the new quantum tomographic method by the rotational wavepacket of a laser-aligned molecule. We also illustrate the power of the new method by applying it to a randomly chosen density matrix rather than that in the laser-aligned case. The iterative QT algorithm also converges after about 20 iterations and density matrix is recovered with considerable accuracy. The density operator of the state to be recovered is set to be

$$
\begin{aligned}
\hat{\rho} = & \frac{2}{21}|00\rangle\langle 00| + \frac{3}{14}|10\rangle\langle 10| + \frac{1}{42}|20\rangle\langle 20| \\
& + \left(\frac{1}{7}|00\rangle\langle 10| + \frac{1}{21}|00\rangle\langle 20| + \frac{1}{14}|10\rangle\langle 20| + \text{H.c.}\right) \\
& + \frac{1}{21}|11\rangle\langle 11| + \frac{3}{28}|21\rangle\langle 21| + \frac{1}{84}|31\rangle\langle 31| \\
& + \left(\frac{1}{14}|11\rangle\langle 21| + \frac{1}{42}|11\rangle\langle 31| + \frac{1}{28}|21\rangle\langle 31| + \text{H.c.}\right) \\
& + \frac{1}{21}|1,-1\rangle\langle 1,-1| + \frac{3}{28}|2,-1\rangle\langle 2,-1| + \frac{1}{84}|3,-1\rangle\langle 3,-1| \\
& + \left(\frac{1}{14}|1,-1\rangle\langle 2,-1| + \frac{1}{42}|1,-1\rangle\langle 3,-1| + \frac{1}{28}|2,-1\rangle\langle 3,-1| + \text{H.c.}\right) \\
& + \frac{1}{21}|22\rangle\langle 22| + \frac{3}{28}|32\rangle\langle 32| + \frac{1}{84}|42\rangle\langle 42| \\
& + \left(\frac{1}{14}|22\rangle\langle 32| + \frac{1}{42}|22\rangle\langle 42| + \frac{1}{28}|32\rangle\langle 42| + \text{H.c.}\right) \\
& + \frac{1}{21}|2,-2\rangle\langle 2,-2| + \frac{3}{28}|3,-2\rangle\langle 3,-2| + \frac{1}{84}|4,-2\rangle\langle 4,-2| \\
& + \left(\frac{1}{14}|2,-2\rangle\langle 3,-2| + \frac{1}{42}|2,-2\rangle\langle 4,-2| + \frac{1}{28}|3,-2\rangle\langle 4,-2| + \text{H.c.}\right) .
\end{aligned} \tag{32}
$$

We impose the error functions of density matrix and probability distribution to measure the accuracy of iteration results, which are defined by

$$\epsilon_n(\hat{\rho}) = \frac{\sum_{J_1 m_1 J_2 m_2} |\langle J_1 m_1|\hat{\rho}|J_2 m_2\rangle_n - \langle J_1 m_1|\hat{\rho}|J_2 m_2\rangle_0|}{\sum_{J_1 m_1 J_2 m_2} |\langle J_1 m_1|\hat{\rho}|J_2 m_2\rangle_0|} \tag{33}$$

$$\epsilon_n(\mathrm{Pr}) = \frac{\sum_{i,j,k} |\mathrm{Pr}_n(\theta_i, \phi_j, t_k) - \mathrm{Pr}_0(\theta_i, \phi_j, t_k)|}{\sum_{i,j,k} |\mathrm{Pr}_0(\theta_i, \phi_j, t_k)|} \tag{34}$$

where the subscript n represents the result of n-th iteration, and 0 represents the correct result.

In Fig. 5 we show the result of identical algorithm given in Fig. 1 and Fig. 2, only with smaller order $J_{\max}$ of density matrix to be recovered. The initial state is given by correct diagonal elements of density matrix. The iteration converged to the expected result with error $\epsilon_{20}(\hat{\rho}) = 3.5 \times 10^{-3}$ and $\epsilon_{20}(\mathrm{Pr}) = 1.7 \times 10^{-3}$.

Especially, we show with the proof-of-principle example that this iterative QT algorithm is insensitive with the initial guess of density matrix. The rotational temperature which provides much information such as initial guess and partial trace, is actually not indispensable to the QT method. Assume we are dealing with a pure QT problem without any additional knowledge to the density matrix to be recovered. As is shown in Fig. 6, a random initial guess will also lead to a converged result after about 30 iterations with error $\epsilon_{30}(\hat{\rho}) = 3.9 \times 10^{-2}$ and $\epsilon_{30}(\mathrm{Pr}) = 9.0 \times 10^{-3}$.

SUPPLEMENTARY NOTE 8. VIBRATIONAL QUANTUM TOMOGRAPHY

Vibrational quantum tomography recovers the density matrix of N vibrational modes from the probability distribution evolution $\mathrm{Pr}(x_1, x_2, \cdots, x_N, t)$

$$\mathrm{Pr}(x_1, x_2, \cdots, x_N, t) = \sum_{\{m_i\}_{i=1}^N} \sum_{\{n_i\}_{i=1}^N} \langle n_1 n_2 \cdots n_N|\hat{\rho}|m_1 m_2 \cdots m_N\rangle \times \prod_{i=1}^{N} \phi_{n_i}(x_i)\phi^*_{m_i}(x_i) e^{i(m_i - n_i)\omega_i t} \,. \tag{35}$$

where $\phi_{n_i}(x_i)$ is the harmonic oscillator wavefunction of the i-th vibrational mode with energy eigenvalue $(n_i + \frac{1}{2})\omega_i$. The dimension problem arises naturally. Here the probability is $(N+1)$-dimensional and density matrix is $2N$-dimensional, which is inadmissible for analytical solutions when $N > 1$. In conventional QT method that is based on integral transform, the orthogonal properties cancel out one summation by integrating over one parameter. For example,

$$\frac{1}{T}\int_0^T dt e^{i(m-n)r\omega_0 t} e^{-ik\omega_0 t} = \delta_{(m-n)r,k} \,, \tag{36}$$

where $T = \frac{2\pi}{\omega_0}$. $f_{mn}(x)$ is the sampling function [12] defined by

$$f_{mn}(x) = \frac{\partial}{\partial x}[\phi_m(x)\varphi_n(x)]\,, \tag{37}$$

where $\phi_m(x)$ and $\varphi_n(x)$ are respectively regular and irregular wavefunctions of harmonic oscillator. The bi-orthogonal properties of sampling function is

$$\int_{-\infty}^{+\infty} dx f_{mn}(x)\phi^*_{m'}(x)\phi_{n'}(x) = \delta_{mm'}\delta_{nn'}\,, \tag{38}$$

under frequency constraints $m - n = m' - n'$.

Our theory, based on the following two procedures, fully utilizes the above orthogonal properties and imposes constraints for lack of dimension. First, we set up the transformation between probability and density matrix in a subspace

$$\begin{aligned} \mathrm{Pr}_{\Delta_1,\Delta_2,\cdots,\Delta_N}(x_1, x_2, \cdots, x_N) &= \sum_{\{m_i\}_{i=1}^N} \sum_{\{n_i\}_{i=1}^N} \langle n_1 n_2 \cdots n_N|\hat{\rho}|m_1 m_2 \cdots m_N\rangle \\ &\quad \times \prod_{i=1}^N \phi_{n_i}(x_i)\phi^*_{m_i}(x_i)\delta_{m_i-n_i,\Delta_i} \end{aligned} \tag{39}$$

$$\langle n_1 n_2 \cdots n_N|\hat{\rho}|m_1 m_2 \cdots m_N\rangle = \int d^N\mathbf{x}\mathrm{Pr}_{\Delta_1,\Delta_2,\cdots,\Delta_N}(x_1, x_2, \cdots, x_N)\prod_{i=1}^N f_{m_i n_i}(x_i)\,. \tag{40}$$

Second, starting from an initial guess, effective physical constraints can be imposed by iterative projection method to get the converged result. For example, the priori knowledge of density matrix of being Hermitian, positive semidefinite and normalized. The algorithm of vibrational state QT and an example of 2D vibrational quantum tomography is shown in Fig. 7 and Fig. 8. The initial guess is given randomly, and only the probability distribution and general properties of density matrix are imposed as constraints during the iteration algorithm.

Similar to rotational QT, the dimension problem can be reflected by the fact that for

$$\mathrm{Pr}_k(x_1, x_2, \cdots, x_N) = \sum_{\{\Delta_i\}_{i=1}^N} \mathrm{Pr}_{\Delta_1,\Delta_2,\cdots,\Delta_N}(x_1, x_2, \cdots, x_N)\delta_{\sum_{i=1}^N \Delta_i r_i, k}\,, \tag{41}$$

unless only one single combination of $\{\Delta_i\}$ satisfies $\sum_{i=1}^N \Delta_i r_i = k$, there is no direct way to obtain $\mathrm{Pr}_{\Delta_1,\Delta_2,\cdots,\Delta_N}(x_1, x_2, \cdots, x_N)$ from the measured wavepacket density distribution, only their sum can be available through Fourier transform of the measured probability distribution evolution

$$\mathrm{Pr}_k(x_1, x_2, \cdots, x_N) = \frac{1}{T}\int_0^T dt e^{-ik\omega_0 t}\mathrm{Pr}(x_1, x_2, \cdots, x_N, t)\,, \tag{42}$$

where we assume $\omega_i = r_i\omega_0$ (r_i are integers and $T = 2\pi/\omega_0$, r_i's are the set of smallest integers to represent the measured frequencies). In the new iterative QT method for N-dimensional vibrational system, we do not need infinitely long time of measurement anymore, which used to be indispensable to fill the whole space of N-dimensional phases [13] while physically infeasible. Besides, in the new iterative QT method, the ratio of frequencies does not have to be irrational, which is important because in reality N-dimensional vibrational systems with commensurable frequencies are ubiquitous.

The pattern function can be approximated around $x = 0$ as [14]

$$f_{nn} \sim -\frac{2}{\pi}\sin[-\pi(n+1/2) + 2\sqrt{2n+1}x]\,. \tag{43}$$

In order to resolve a period of the oscillation of the pattern function that arises in the convolution (Eq. 40), the required spatial resolution for reconstructing vibrational density matrix up to N-th order has to be better than $\delta x \leq \pi/2\sqrt{2N+1}$. The maximal order of the desired density matrix also sets demand on the temporal resolution. Suppose d time intervals are measured for a half period $T/2 = \pi/\omega_0$. From Eq. 42, we have a phase resolution of $k\pi/d$ for the Fourier transformation of probability distribution function. The aliasing phenomena defines the maximal order of density matrix we can access to be $N = d/k - 1$, thus the required temporal resolution is

$$\delta t \leq \frac{T}{2(N+1)k} \leq \frac{T}{2(N+1)\sum_i r_i}\,. \tag{44}$$

The quantum tomography procedure presented above can be easily generalized to systems with strong anharmonicity , or when coupling among different vibrational modes exist. In general case, the Hamiltonian [15]

$$\hat{H} = \sum_{i=1}^{N}\hat{h}_i(x_i) + V(x_1, x_2, \cdots, x_N)\,, \tag{45}$$

where $\hat{h}_i$ is the separable part for i-th vibrational mode with eigenstate $\phi_{n_i}(x_i)$, and $V(x_1, x_2, \cdots, x_N)$ is coupling potential among N vibrational modes. The eigenstate is a linear combination of product 1D wavefunctions assigned with quantum numbers $I = \{I_1, I_2, \cdots, I_N\}$ with energy eigenvalue E_I

$$\Psi_I(x_1, x_2, \cdots, x_N) = \sum_{i_1, i_2, \cdots, i_N} C_I^{i_1, i_2, \cdots, i_N}\prod_{\alpha=1}^{N}\phi_{i_\alpha}(x_\alpha)\,. \tag{46}$$

The wavefunction ansatz and the self-consistent field state interaction (SCF-SI) method can be extended to the treatment of molecular vibrations with anharmonic potential and strong coupling.

To solve the vibrational Hamiltonian efficiently for this general case, the separable part Hamiltonian $\hat{h}_i$ for the anharmonic vibrational modes can be replaced by, for example, Morse potential model and corresponding pattern function [16] can be used. The iterative projection algorithm for quantum tomography should be set up based on the transformation between probability and density matrix in a subspace

$$\mathrm{Pr}_{\Delta_1,\Delta_2,\cdots,\Delta_N}(x_1,x_2,\cdots,x_N) = \sum_{I,J}\langle I|\hat{\rho}|J\rangle \sum_{i_1,i_2,\cdots,i_N}\sum_{j_1,j_2,\cdots,j_N} C_I^{i_1,i_2,\cdots,i_N} C_J^{j_1,j_2,\cdots,j_N *} \times \prod_{\alpha=1}^{N} \phi_{i_\alpha}(x_\alpha)\phi^*_{j_\alpha}(x_\alpha)\delta_{\omega_{i_\alpha}-\omega_{j_\alpha},\omega_{\Delta_\alpha}} \tag{47}$$

$$\int d^N\mathbf{x}\mathrm{Pr}_{\Delta_1,\Delta_2,\cdots,\Delta_N}(x_1,x_2,\cdots,x_N)\prod_{\alpha=1}^{N} f_{i_\alpha j_\alpha}(x_\alpha) = \sum_{I,J}\langle I|\hat{\rho}|J\rangle C_I^{i_1,i_2,\cdots,i_N} C_J^{j_1,j_2,\cdots,j_N *}\,. \tag{48}$$

where the frequency constraint of sampling function requires $\omega_{i_\alpha}-\omega_{j_\alpha}=\omega_{\Delta_\alpha}$ $(\alpha=1,2,\cdots,N)$. The density matrix element can be solved from the linear equation of 48. If there are n basis eigenstate for i-th uncoupled vibrational mode $\phi_{n_i}(x_i)$, the coupled density matrix can be recovered to the order of $(2n)^{N/2}$. Similarly, the procedure starts from an initial guess and imposes constraints to both density matrix space and probability space. Besides basic properties of density matrix and probability distribution, the subspace probability should also satisfy

$$\begin{aligned} \mathrm{Pr}_{\omega_{IJ}}(x_1,x_2,\cdots,x_N) &= \frac{1}{T}\int_0^T dt\mathrm{Pr}(x_1,x_2,\cdots,x_N,t)e^{-i\omega_{IJ}t} \\ &= \sum_{\omega_I-\omega_J=\omega_{IJ}}\langle I|\hat{\rho}|J\rangle\varphi_{i_1,i_2,\cdots,i_N}(x_1,x_2,\cdots,x_N)\varphi^*_{j_1,j_2,\cdots,j_N}(x_1,x_2,\cdots,x_N) \\ &= \sum_{\Delta_1,\Delta_2,\cdots,\Delta_N}\mathrm{Pr}_{\Delta_1,\Delta_2,\cdots,\Delta_N}(x_1,x_2,\cdots,x_N)\delta_{\omega_I-\omega_J,\omega_{IJ}}\,. \end{aligned} \tag{49}$$

where ω_I and ω_J are energy eigenvalues of the coupled Hamiltonian, T is the common period for all vibrational frequency intervals.

To enhance the convergence of iterative QT procedure for vibrational states, physical constraints can be imposed on the diagonal matrix elements of the density matrix, which is experimentally accessible, e.g. through photoelectron spectra and absorption spectra, which can directly provide constraints on diagonal density matrix elements of basis states with eigenenergy E [17].

As a final remark, for vibrational QT, it is sometimes neccessary to use the velocities of nuclei as constraining physical conditions, in the case that the basis states of density matrix is energetically degenerate. For example, given the ratio of two vibrational frequencies $r_1/r_2 = 1/2$, consider a mixed state consisting of $|20\rangle$ and $|10\rangle$ (the pure state is a special case of it), their

density matrix is

$$\rho = \begin{pmatrix} \langle 20|\hat{\rho}|20\rangle & \langle 20|\hat{\rho}|01\rangle \\ \langle 01|\hat{\rho}|20\rangle & \langle 01|\hat{\rho}|01\rangle \end{pmatrix} = \begin{pmatrix} \rho_{11} & \rho_{12} \\ \rho_{21} & \rho_{22} \end{pmatrix}. \tag{50}$$

The probability distribution

$$\begin{aligned} \mathrm{Pr}(x_1, x_2, t) &= \rho_{11}\phi_2^2(x_1)\phi_0^2(x_2) + \rho_{22}\phi_0^2(x_1)\phi_1^2(x_2) \\ &\quad + (\rho_{12}+\rho_{21})\phi_2(x_1)\phi_0(x_2)\phi_0(x_1)\phi_1(x_2) \end{aligned} \tag{51}$$

could not reflect the imaginary part of the off-diagonal density matrix elements because the degeneracy of the two basis states smears out the temporal evolution of the probability distribution. If $|20\rangle$ and $|01\rangle$ belong to the same symmetry representation, their coupling will lead to Fermi resonance and the degeneracy can be lifted. In the case that $|20\rangle$ and $|01\rangle$ are exactly degenerate, additional constraints must be imposed. Because with the ultrafast diffraction method, the velocity of nuclei and thus their momenta can be extracted experimentally, we can naturally construct physical constraints through products of momenta, such as $p_{x_1}^2 p_{x_2}$, since

$$\mathbf{A} = (\hat{p}_{x_1}^2 \hat{p}_{x_2}) = \begin{pmatrix} a_{11} & a_{12} \\ a_{21} & a_{22} \end{pmatrix} \tag{52}$$

has nonzero imaginary part of non-diagonal matrix elements. For example,

$$\begin{aligned} a_{12} &= \int dx_1 \phi_2(x_1)\left(-\frac{\partial^2}{\partial x_1^2}\right)\phi_0(x_1)\int dx_2\phi_0(x_2)\left(-i\frac{\partial}{\partial x_2}\right)\phi_1(x_2) \\ &= \int_{-\infty}^{\infty} dx_1 \frac{1}{\pi^{1/4}}\sqrt{\frac{\alpha_1}{2}}(2\alpha_1^2 x_1^2 - 1)e^{-\frac{1}{2}\alpha_1 x_1^2}\frac{\partial^2}{\partial x_1^2}\left(\frac{\sqrt{\alpha_1}}{\pi^{1/4}}e^{-\frac{1}{2}\alpha_1 x_1^2}\right) \\ &\times \int_{-\infty}^{\infty} dx_2 \frac{\sqrt{\alpha_2}}{\pi^{1/4}}e^{-\frac{1}{2}\alpha_2 x_2^2}\frac{\partial}{\partial x_2}\left(\frac{\sqrt{2\alpha_2}}{\pi^{1/4}}e^{-\frac{1}{2}\alpha_2 x_2^2}\right) = -i\frac{\alpha_1^2\alpha_2}{2} \\ a_{21} &= a_{12}^* = i\frac{\alpha_1^2\alpha_2}{2} \end{aligned} \tag{53}$$

The observable

$$\begin{aligned} \langle\hat{A}\rangle &= m_1^2 v_1^2 m_2 v_2 = \mathrm{Tr}(\hat{\rho}\hat{A}) \\ &= \rho_{11}a_{11} + \rho_{12}a_{21} + \rho_{21}a_{12} + \rho_{22}a_{22} \\ &= \rho_{11}a_{11} + \rho_{22}a_{22} + 2\mathrm{Re}[\rho_{12}a_{21}] \\ &= \rho_{11}a_{11} + \rho_{22}a_{22} - \alpha_1^2\alpha_2\mathrm{Im}[\rho_{12}] \end{aligned} \tag{54}$$

contains information of imaginary part of non-diagonal density matrix elements $\mathrm{Im}[\rho_{12}] = -\mathrm{Im}[\rho_{21}]$, with which we can effectively determine the imaginary part of the off-diagonal density

matrix elements between exactly degenerate basis states, by using the products of velocities as physical constraints in the iterative QT procedure.

Throughout the paper, we focus on recovering the density matrix, which is interconnected with the Wigner function $W(q,p)$ via the overlapping formula,

$$\begin{aligned}\rho_{mn} &= \mathrm{Tr}[\hat{\rho}|n\rangle\langle m|] \\ &= \frac{1}{2\pi}\int_{-\infty}^{\infty} dq \int_{-\infty}^{\infty} dp W(q,p) W_{|n\rangle\langle m|}(q,p)\,,\end{aligned} \tag{55}$$

where $W_{\hat{\mathcal{O}}}(q,p) = (1/2\pi)\int dx \exp(-ipx)\langle q-\frac{x}{2}|\hat{\mathcal{O}}|q+\frac{x}{2}\rangle$. Especially, the Wigner function can be expressed in terms of the density operator $\hat{\rho}$ as $W(q,p) = W_{\hat{\rho}}(q,p)$.

SUPPLEMENTARY REFERENCES

[1] Pabst, S., Ho, P. J. & Santra, R. Computational studies of X-ray scattering from three-dimensionally-aligned asymmetric-top molecules. *Phys. Rev. A* **81**, 043425 (2010).

[2] Stapelfeldt, H. & Seideman, T. Colloquium: Aligning molecules with strong laser pulses. *Rev. Mod. Phys.* **75**, 543–557 (2003).

[3] Zandi, O., Wilkin, K. J., Xiong, Y. & Centurion, M. High current table-top setup for femtosecond gas electron diffraction. *Struct. Dynamics* **4**, 044022 (2017).

[4] Xiong, Y., Wilkin, K. J. & Centurion, M. High-resolution movies of molecular rotational dynamics captured with ultrafast electron diffraction. *Phys. Rev. Research* **2**, 043064 (2020).

[5] Holmes, T. J. *et al.* Light microscopic images reconstructed by maximum likelihood deconvolution. In *Handbook of biological confocal microscopy*, 389–402 (Springer, 1995).

[6] Biggs, D. S. & Andrews, M. Acceleration of iterative image restoration algorithms. *Appl. Optics* **36**, 1766–1775 (1997).

[7] Jansson, P. A. *Deconvolution of images and spectra* (Academic Press, 1997).

[8] Mouritzen, A. S. & Mølmer, K. Quantum state tomography of molecular rotation. *J. Chem. Phys.* **124**, 244311 (2006).

[9] Dooley, P. W. *et al.* Direct imaging of rotational wave-packet dynamics of diatomic molecules. *Phys. Rev. A* **68**, 234061–2340612 (2003).

[10] Ho, P. J. & Santra, R. Theory of X-ray diffraction from laser-aligned symmetric-top molecules. *Phys. Rev. A* **78**, 053409 (2008).

[11] Ischenko, A. A. & Aseyev, S. A. *Time-resolved Electron Diffraction: For Chemistry, Biology and Materials Science*, vol. 184 of *Advances in Imaging and Electron Physics* (Academic Press, 2014).

[12] Leonhardt, U. & Raymer, M. G. Observation of moving wave packets reveals their quantum state. *Phys. Rev. Lett.* **76**, 1985 (1996).

[13] Mouritzen, A. S. & Mølmer, K. Tomographic reconstruction of quantum states in N spatial dimensions. *Phys. Rev. A* **73**, 042105 (2006).

[14] Leonhardt, U., Munroe, M., Kiss, T., Richter, T. & Raymer, M. Sampling of photon statistics and density matrix using homodyne detection. *Opt. Commun.* **127**, 144–160 (1996).

[15] Bowman, J. M., Christoffel, K. & Tobin, F. Application of SCF-SI theory to vibrational motion in polyatomic molecules. *J. Phys. Chem.* **83**, 905–912 (1979).

[16] Leonhardt, U. State reconstruction of anharmonic molecular vibrations: Morse-oscillator model. *Phys. rev. A* **55**, 3164–3172 (1997).

[17] Heller, E. J. Quantum corrections to classical photodissociation models. *J. Chem. Phys.* **68**, 2066–2075 (1978).
